# Enhancing vascular access planning in CKD: validating the 40% KFRE threshold for predicting ESKD in a French retrospective cohort study

**DOI:** 10.1093/ckj/sfae220

**Published:** 2024-07-13

**Authors:** Maxime Ingwiller, Nicolas Keller, Thierry Krummel, Eric Prinz, Lydie Steinmetz, Thierry Hannedouche, Nans Florens

**Affiliations:** Nephrology Department, CHU de Strasbourg, Service de Néphrologie, CHU de Strasbourg, Strasbourg, France; Dialysis Center, AURAL Strasbourg, Strasbourg, France; Nephrology Department, CHU de Strasbourg, Service de Néphrologie, CHU de Strasbourg, Strasbourg, France; Nephrology Department, CHU de Strasbourg, Service de Néphrologie, CHU de Strasbourg, Strasbourg, France; Nephrology Department, CHU de Strasbourg, Service de Néphrologie, CHU de Strasbourg, Strasbourg, France; Vascular Surgery Department, CHU de Strasbourg, Service de Néphrologie, CHU de Strasbourg, Strasbourg, France; Dialysis Center, AURAL Strasbourg, Strasbourg, France; Nephrology Department, CHU de Strasbourg, Service de Néphrologie, CHU de Strasbourg, Strasbourg, France; UMR INSERM 1109, Molecular Immuno-Rhumatology, Translational Medicine Federation of Strasbourg (FMTS), Faculty of Medicine, University of Strasbourg, Strasbourg, France; INI-CRCT (Cardiovascular and renal trialists), F-CRIN Network

**Keywords:** chronic kidney disease, clinical decision-making, dialysis preparation, end-stage kidney disease prediction, vascular access planning

## Abstract

**Background:**

Establishing the optimal timing for creating vascular access in patients with chronic kidney disease (CKD) is a critical and challenging aspect of patient management. The Kidney Disease: Improving Global Outcomes guidelines propose using a 40% 2-year threshold based on the Kidney Failure Risk Equation (KFRE) for this purpose. However, the effectiveness of this threshold compared with traditional methods, such as estimated glomerular filtration rate (eGFR), is not well-established.

**Methods:**

In this monocentric retrospective cohort study, we analyzed data from patients referred for vascular mapping before arteriovenous fistula (AVF) creation between April 2013 and June 2023. The study aimed to compare the ≥40% 2-year KFRE threshold with a <15 mL/min/1.73 m² eGFR threshold for predicting end-stage kidney disease (ESKD). We assessed the probability of ESKD, considering death before AVF creation as a competing risk. Discrimination between KFRE and eGFR was evaluated using C-statistics.

**Results:**

The study included 238 patients with a mean age of 65.2 years and a mean eGFR of 13.3 mL/min/1.73 m². Over a median follow-up of 10.7 months, 178 patients developed ESKD, and 21 died before ESKD. Probability of ESKD at 1 year was 77.6% (95% CI 69.9%–85.3%) using a ≥40% 4-variable KFRE threshold versus 65.8% (95% CI 58.3%–73.3%) using a <15 mL/min/1.73 m² eGFR threshold. The C-statistics indicated better predictive ability for the 8-variable KFRE at 6 months [0.82 (95% CI 0.76–0.88)], while both 4- and 8-variable KFRE models were effective for 1-year predictions [0.835 (95% CI 0.78–0.89) and 0.82 (95% CI 0.76–0.875), respectively]. Sensitivity and specificity analyses favored the ≥40% KFRE threshold over the eGFR threshold.

**Conclusions:**

This study suggests that using a ≥40% 2-year KFRE threshold for planning vascular access in CKD patients is promising and potentially superior to the traditional <15 mL/min/1.73 m² eGFR threshold. This approach may offer a balance between minimizing premature AVF creation and the risk of starting dialysis via a central venous catheter.

## Graphical Abstract

**Graphical Abstract:**
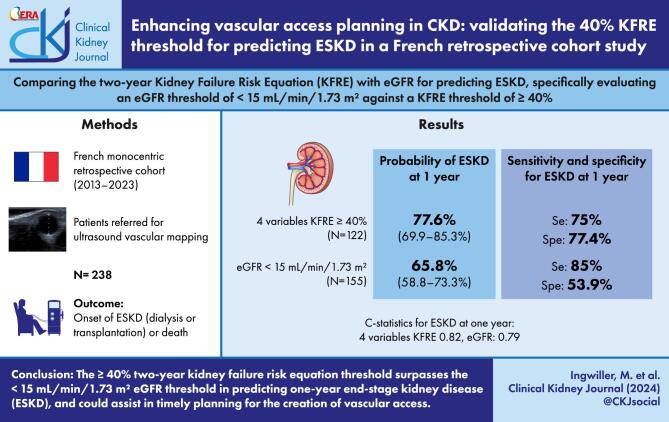

KEY LEARNING POINTS **What was known:**

- Prior research highlighted the importance of timely vascular access creation for dialysis in chronic kidney disease (CKD) patients, but optimal timing remained ambiguous.
- The Kidney Failure Risk Equation (KFRE) had been suggested as a predictive tool, yet its efficacy in specific clinical settings like vascular access planning was not well-established.
- There was a growing need to refine pre-dialysis interventions, balancing the risks and benefits of early versus late vascular access creation.

**This study adds:**

- Our study demonstrates that a ≥40% 2-year KFRE threshold is potentially more effective than the traditional <15 mL/min/1.73 m² eGFR threshold for planning vascular access in CKD patients.
- The findings suggest a balance between minimizing premature arteriovenous fistula (AVF) creation and the risk of initiating dialysis via a central venous catheter.
- It highlights the superior predictive capability of the 8-variable KFRE over the 4-variable model for predicting 6-month end-stage kidney disease (ESKD), offering new insights into patient-specific management strategies.

**Potential impact:**

- This study could influence clinical guidelines by integrating the ≥40% 2-year KFRE threshold into vascular access planning, leading to more personalized and effective CKD management.
- By validating the use of KFRE in a clinical setting, it may encourage more nephrologists to adopt this tool for better decision-making regarding dialysis preparations.
- Use of 8-variable KFRE could help in gaining precision for predicting ESKD, especially at 6 months.
- The findings may prompt policy changes in CKD management, emphasizing risk stratification and tailored patient care to improve outcomes and reduce healthcare burdens.

## INTRODUCTION

Determining the optimal timing for the creation of dialysis vascular access presents a significant challenge. Despite concerted efforts by clinicians, data from the 2021 French Renal Epidemiology and Information Network (REIN) Registry [1] reveal that 58% of patients started dialysis using a central venous catheter. Conversely, early creation of vascular access risks exposing patients to the heightened possibility of mortality [2, 3] prior to the initiation of dialysis. Initiating dialysis more than 6–9 months following the creation of vascular access is associated with increased rates of post-creation procedures, without improving the likelihood of starting dialysis via an arteriovenous fistula (AVF) [4].

Recent guidelines from the Kidney Disease Outcomes Quality Initiative (KDOQI) advise that referrals for vascular access creation should be made when a patient's estimated glomerular filtration rate (eGFR) falls between 15 and 20 mL/min/1.73 m², particularly in those exhibiting a progressive decline in kidney function [5]. However, relying solely on eGFR for predicting hemodialysis initiation within a 2-year timeframe is insufficient; other critical factors, including the extent of proteinuria and the trajectory of eGFR decline, must also be considered [6].

The Kidney Failure Risk Equation (KFRE) [7, 8], which employs a 4-variable equation incorporating eGFR, age, sex and urinary albumin-to-creatinine ratio (UACR), is the most prevalent model for identifying patients at elevated risk of end-stage kidney disease (ESKD). This model estimates the risk of ESKD over 2 and 5 years. An expanded equation including calcium, phosphate, bicarbonate and albumin did not demonstrate enhanced predictive accuracy [7]. The efficacy of KFRE in advanced chronic kidney disease (CKD) is well-validated across various kidney disorders [9].

The 2024 CKD Kidney Disease: Improving Global Outcomes (KDIGO) guidelines recommend a 5-year risk of 3%–5% for nephrology referral and a 2-year risk of 40% for planning vascular access or transplantation referral [10]. Although the 5-year risk threshold has been both retrospectively and prospectively validated [11], the application of a 2-year risk threshold of 40% for vascular access planning remains under-explored. In a prior study, we discovered that employing a 2-year risk threshold of ≥40% using KFRE showed comparable sensitivity and specificity to an eGFR threshold of <15 mL/min/1.73 m² in predicting ESKD [12], despite some methodological limitations. Another recent study, utilizing a parametric survival model to estimate the time to kidney failure, indicated that for patients with an eGFR <15 mL/min/1.73 m^2^ or a KFRE 2-year risk >40%, both KFRE risk and eGFR were similarly predictive of time to kidney failure [13]. However, this study did not exclusively focus on patients with advanced CKD and did not account for the competing risk of death.

On the other hand, a recently published retrospective cohort study from British Columbia, Canada, found that incorporating the KFRE into existing eGFR-based referral practices for vascular access creation can improve outcomes. More patients started dialysis with an arteriovenous fistula or graft, and fewer patients had vascular access that remained unused for extended periods [14]. However, this study did not evaluate the 8-variable KFRE and did not provide data on eGFR at dialysis initiation. Additionally, practices concerning dialysis initiation may differ between countries.

The objective of our study was to compare the predictive abilities of the 2-year 4-variable and 8-variable KFRE and eGFR alone for forecasting ESKD in patients with CKD stages 4 and 5 who were referred for ultrasound vascular mapping for AVF creation. Specifically, we sought to evaluate the effectiveness of an eGFR threshold of <15 mL/min/1.73 m² and a KFRE threshold of ≥40%.

## MATERIALS AND METHODS

### Study population

Our research involved a retrospective, single-center cohort study of patients referred for ultrasound vascular mapping at Hôpitaux Universitaires de Strasbourg, France from April 2013 to June 2023. In our institution, patients first receive a referral for vascular mapping from their primary nephrologist, followed by scheduling of AVF creation.

Dates and results of vascular mapping were recorded in a file used for selecting patients, with the date of vascular mapping serving as the study index date. We then retrospectively collected the clinical and biological data.

The follow-up for patients continued until the onset of ESKD—marked by dialysis or kidney transplantation—or in cases of death, loss to follow-up, or end of data collection. Inclusion required a minimum of 1 year of follow-up if no aforementioned events occurred. We excluded from our study patients who had previously undergone kidney transplantation, those already on dialysis and individuals <18 years of age.

### Variables

The data gathered included: patient age, underlying nephropathy, plasma creatinine, eGFR, UACR or urine protein-to-creatinine ratio (UPCR), serum levels of calcium, phosphate, bicarbonate, albumin, and dates of vascular mapping, AVF creation, ESKD occurrence or death. Also recorded were the eGFR at the time of dialysis initiation or kidney transplantation, the type of access used at dialysis initiation (central venous catheter, AVF or peritoneal dialysis), and the reasons for initiating dialysis.

Biological variables were collected at the time of vascular mapping with a permissible variation of ±3 months, except for UACR, for which we considered the most recent value within the preceding 365 days, in line with prior studies [7, 13].

The eGFR was calculated using the CKD Epidemiology Collaboration equation [15]. The UACR was quantified in mg/g. When only UPCR was available, we applied a conversion factor as delineated by Tangri *et al*. (UPCR in mg/g multiplied by 0.569 for females and 0.376 for males to convert to UACR in mg/g) [7, 8].

The 2-year risk for KFRE, both 4-variable and 8-variable, was calculated according to the Calibrated—non-North American equations [8]. There were no instances of missing data for the 4-variable KFRE and only two instances for the 8-variable KFRE, leading us to conduct a complete case analysis without any data imputation.

### Statistical analysis

Our statistical analysis commenced with the description of quantitative variables, employing mean and 95% confidence intervals (95% CI) for normally distributed data, and median with interquartile range (IQR) for non-normally distributed data. Qualitative variables were summarized using counts and percentages. Time to event data were presented as median and IQR. The cumulative incidence function (CIF) for ESKD and death, based on a <15 mL/min/1.73 m² eGFR threshold or a ≥40% KFRE threshold, were estimated using the “cuminc()” function in the “cmprsk” package, specifically designed for competing risk data in R.

To evaluate the predictive performance of eGFR, 4-variable KFRE and 8-variable KFRE in forecasting ESKD at 6 months, 1 year and 2 years, time-dependent receiver operating characteristic (ROC) curves were generated. The discriminative ability was quantified using C-statistics, representing the area under the ROC curve, at each time point. These analyses were conducted using the “timeROC” package in R, which accommodates censored data with competing risks [16].

The calibration of the 4-variable KFRE for predicting 2-year ESKD risk was assessed graphically using a locally estimated scatterplot smoothing (loess) calibration curve from the “CalibrationCurves” package in R [17].

We computed the sensitivity, specificity and Youden Indexes at 6 months, 1 year and 2 years for a <15 mL/min/1.73 m² eGFR threshold, and for ≥40% thresholds of both the 4-variable and 8-variable KFRE. The Youden Index, which integrates sensitivity and specificity into a single metric (calculated as: sensitivity + specificity – 1), ranges from 0 to 1, with a perfect test scoring 1.

Additionally, we employed Net Benefit Decision Curves for comparing the net benefits of the 4-variable and 8-variable KFRE at 6 months and 1 year. The net benefit is computed as the true positives minus the false positives, weighted by the sample population, across a predefined range of threshold probabilities. The formula for net benefit [18], where Pt denotes the threshold probability, is as follows:


\begin{eqnarray*}Net\ \textit{benefit} = \frac{{\textit{true}\ \textit{positives}}}{{\textit{total}\ \textit{population}}} - \frac{{\textit{false}\ \textit{positives}}}{{\textit{total}\ \textit{population}}} \times \frac{{{{p}_t}}}{{1 - {{p}_t}}}
\end{eqnarray*}

This net benefit is plotted on the y-axis of a decision curve analysis, with the range of threshold probabilities on the x-axis. Interpretation of these curves involves comparing the net benefits between the models and against the default strategies of either intervening on all or none of the patients for a given threshold probability.

Our paper adheres to the TRIPOD guidelines (Transparent Reporting of a Multivariable Prediction Model for Individual Prognosis or Diagnosis) [19]. All statistical analyses were conducted using R software (v4.3.0; R Core Team 2023).

## RESULTS

Our study included 238 patients, with a median follow-up duration of 10.7 months (range 2 days to 140 months, IQR 4.2–19.8 months).

### Patient characteristics and clinical outcomes

Table 1 presents the primary characteristics of the patient cohort. The average age was 65.2 years (95% CI 63.4–67 years), the mean eGFR was 13.3 mL/min/1.73 m² (95% CI 12.8–13.9 mL/min/1.73 m²) and the mean UACR was 1517 mg/g (95% CI 1306–1728 mg/g). Notably, there were no significant metabolic complications related to CKD.

**Table 1:** Initial clinical and biological characteristics of patients.

Variables	
Age, years	65.2 (63.4–67)
Male sex, *n* (%)	150 (63)
UACR (mg/g), mean (95% CI)	1517 (1306–1728)
UACR (mg/g), median (IQR)	957.5 (264–2260)
eGFR (mL/min/1.73 m²), mean (95% CI)	13.3 (12.8–13.9)
eGFR (mL/min/1.73 m²), median (IQR)	13 (10.2–16)
eGFR (mL/min/1.73 m²), min–max	3–37
Serum albumin (g/L)^a^	38.3 (37.7–39)
Serum bicarbonate (mmol/L)^a^	23.1 (22.6–23.5)
Serum calcium (mmol/L)^a^	2.22 (2.19–2.24)
Serum phosphate (mmol/L)^a^	1.54 (1.49–1.6)
4-variable KFRE (%), mean (95% CI)	42.6 (39.3–45.8)
4-variable KFRE (%), median (IQR)	39.3 (21.2–61.7)
8-variable KFRE (%)^a^, mean (95% CI)	45.9 (42–49.9)
8-variable KFRE (%)^a^, median (IQR)	37.7 (19.5–74.4)
Causal nephropathy, *n* (%)	
Diabetes	93 (39.1)
Other glomerular diseases	40 (16.8)
Vascular diseases	42 (15.5)
Tubulointerstitial diseases	34 (14.3)
Cystic and congenital diseases	18 (7.6)
Other	7 (2.9)
Unknown	4 (1.7)

Data are presented as counts (percentage) for categorical variables and mean (95% CI of the mean) or median (IQR) for continuous variables.

^a^Missing data = 2.

The mean 4-variable KFRE was 42.6% (95% CI 39.3%–45.8%) and the mean for the 8-variable KFRE was 45.9% (95% CI 42%–49.9%). Among the participants, 122 (51.2%) had a 4-variable KFRE ≥40%, and 155 (65.1%) had an eGFR <15 mL/min/1.73 m².

Fifty-one patients (21.4%) with an eGFR <15 mL/min/1.73 m² had a KFRE <40% and 12 patients (5%) with an eGFR ≥15 mL/min/1.73 m² had a KFRE ≥40%.

Of the participants, 178 (74.8%) developed ESKD, 21 (9.2%) died before reaching ESKD and 39 (16.4%) were right-censored, including 17 with a follow-up between 1 and 2 years who experienced no event (Table 2). A total of 196 patients (82.3%) underwent AVF creation, with a median time to first AVF creation of 66 days (IQR 35–132.5 days). Notably, 54 patients (29.7%) had to initiate dialysis via a central venous catheter. The primary reason for dialysis initiation was a low eGFR as determined by the referring nephrologist (61%), with a mean eGFR at initiation of 8.4 mL/min/1.73 m² (95% CI 8–8.8 mL/min/1.73 m²).

**Table 2:** Description of outcomes.

Outcomes	
Observation time in months, median (IQR)	10.7 (4.2–19.8)
AVF creation, *n* (%)	196 (82.3)
Time to first AVF creation in days , median (IQR)	66 (35–132.5)
Death, *n* (%)	21 (9.2)
ESKD, *n* (%)	178 (74.8)
eGFR at ESKD (mL/min/1.73 m²)^a^, mean (95% CI)	8.4 (8–8.8)
eGFR at ESKD (mL/min/1.73 m²)^a^, median (IQR)	8 (7–10)
ESKD modality (*N* = 178), *n* (%)	
Hemodialysis on AVF	115 (64.2)
Hemodialysis on central venous catheter	54 (29.7)
Peritoneal dialysis	3 (1.6)
Kidney transplantation	6 (3.3)
Reasons for starting dialysis (*N* = 172), *n* (%)	
Low eGFR	105 (61)
Electrolytic disorders	13 (7.6)
Uremia	19 (11)
Fluid overload	26 (15)
Acute kidney injury	2 (1.1)
Post-nephrectomy	2 (1.1)
Unknown	5 (2.9)

^a^Missing data = 6.

Of the 83 patients who underwent AVF creation but did not start dialysis on an AVF, 38 (45.8%) initiated dialysis on a central venous catheter (21 before AVF creation and 17 after), 5 (6%) were transplanted, 2 (2.4%) died, 1 (1.2%) was reoriented to peritoneal dialysis, 1 (1.2%) was reoriented to conservative care and 36 (43.4%) did not initiate dialysis by the end of the follow-up period.

### Cumulative incidences

**Figure 1:**
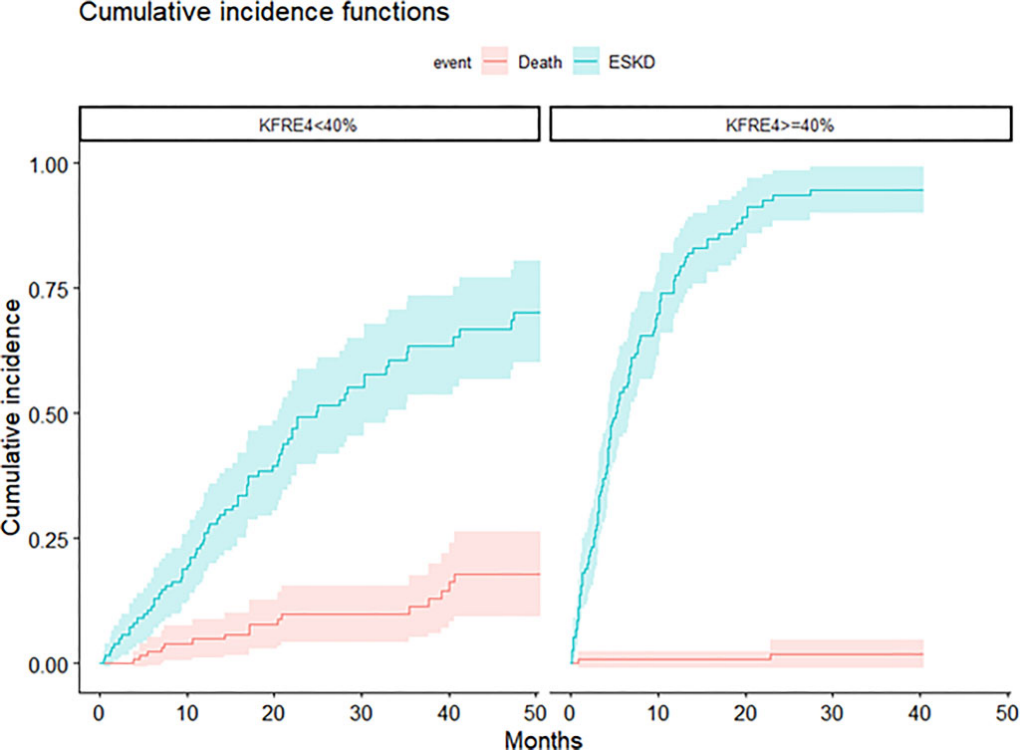
CIF for death and ESKD according to 4-variable KFRE (<40% versus ≥40%).

Figure 1 illustrates the cumulative incidences of death and ESKD for patients with a 4-variable KFRE <40% and ≥40%. For those with a 4-variable KFRE ≥40%, the probability of ESKD was 54.3% (95% CI 45.2%–63.4%) at 6 months, 77.6% (95% CI 69.9%–85.3%) at 12 months and 93.6% (95% CI 88.6%–98.7%) at 24 months. The probability of dying before reaching ESKD remained low overall: 1.96% (95% CI 0%–4.8%) at 24 months. Conversely, for those with a 4-variable KFRE <40%, the competing risk of dying before reaching ESKD was higher: 10% at 24 months (95% CI 4.3%–15.6%).

**Figure 2:**
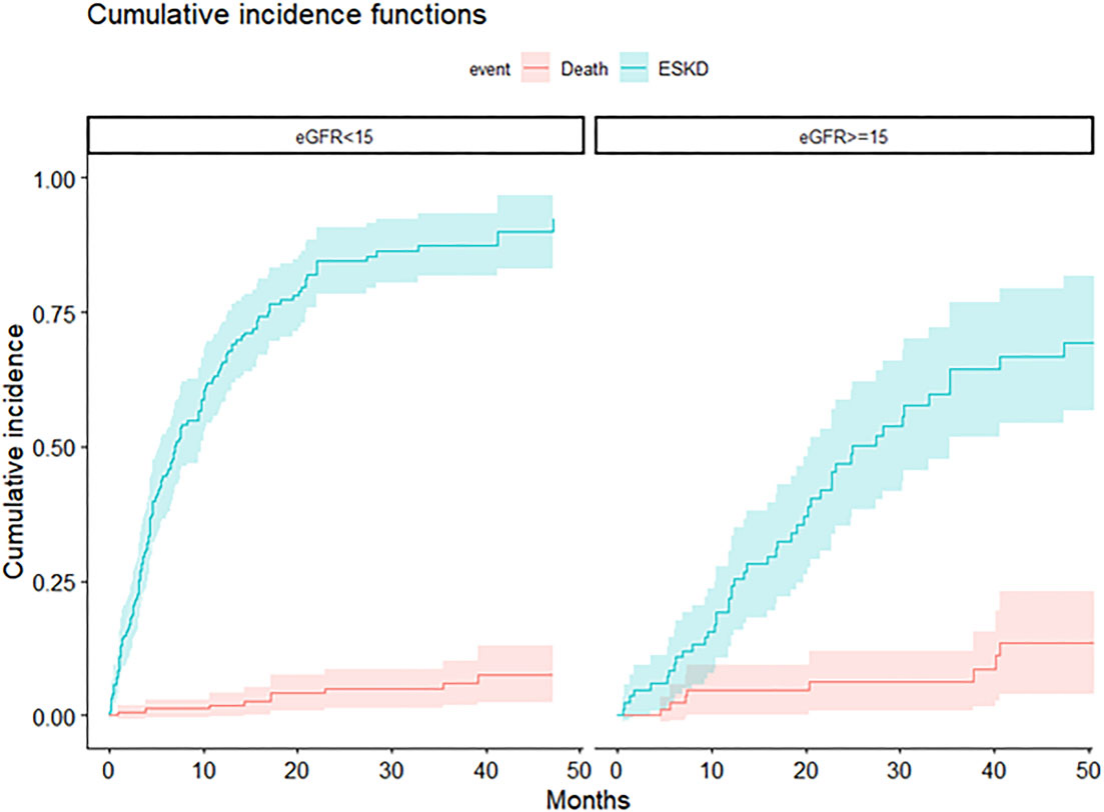
CIF for death and ESKD according to eGFR (<15 versus ≥15 mL/min/1.73 m²).

Figure 2 shows the cumulative incidences of death and ESKD for patients with an eGFR <15 or ≥15 mL/min/1.73 m². For patients with an eGFR <15 mL/min/1.73 m², the probability of ESKD was 44.5% (95% CI 36.7%–52.4%) at 6 months, 65.8% (95% CI 58.3%–73.3%) at 12 months and 83.7% (95% CI 77.5%–90%) at 24 months. The competing risk of dying before ESKD at 24 months was somewhat higher: 5.7% (95% CI 1.8%–9.7%). Supplementary data, Fig. S1 shows the cumulative incidences of death and ESKD for patients with an 8-variable KFRE <40% and ≥40%, indicating that the 8-variable KFRE was marginally more accurate than the 4-variable KFRE in predicting ESKD at 6 and 12 months.

### Discrimination

**Figure 3:**
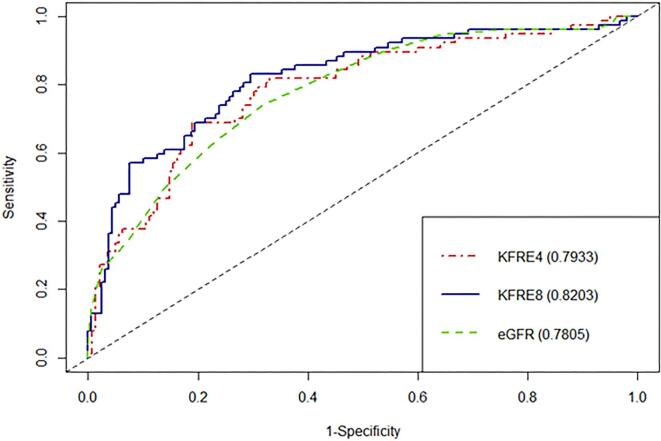
ROC curves and C-statistics for 6 months ESKD according to eGFR, 4-variable KFRE and 8-variable KFRE.

**Figure 4:**
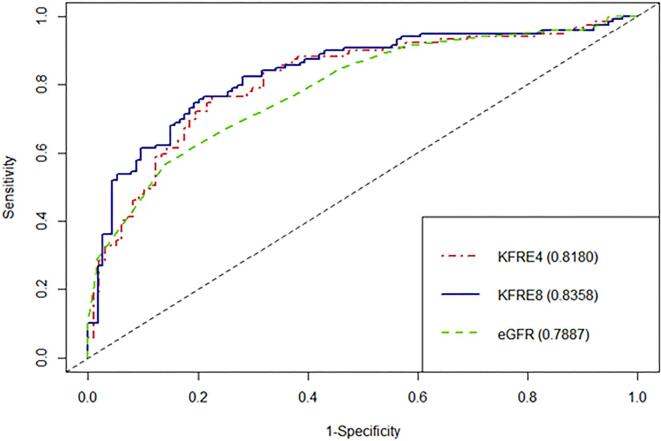
ROC curves and C-statistics for 1 year ESKD according to eGFR, 4-variable KFRE and 8-variable KFRE.

The discrimination abilities of the 4-variable KFRE and eGFR at 6 months were comparable, with C-statistics of 0.79 (95% CI 0.73–0.86) and 0.78 (95% CI 0.72–0.84), respectively. The 8-variable KFRE showed improved discrimination, with a C-statistic of 0.82 (95% CI 0.76–0.88) (Fig. 3). At 1 year, both 4- and 8-variable KFREs showed a trend towards better discrimination than eGFR, with C-statistics of 0.835 (95% CI 0.78–0.89), 0.82 (95% CI 0.76–0.875) and 0.79 (95% CI 0.73–0.85), respectively (Fig. 4). At 2 years, both the 4- and 8-variable KFREs outperformed eGFR, with C-statistics of 0.83 (95% CI 0.76–0.9) and 0.84 (95% CI 0.78–0.90) compared with 0.77 (95% CI 0.69–0.84) for eGFR (Supplementary data, Fig. S2), with only the difference between the 8-variable KFRE and eGFR at 2 years being statistically significant (*P *= .022).

### Calibration

Supplementary data, Fig. S3 presents the flexible calibration curve for the 4-variable KFRE. In our cohort, the risk was clearly underestimated, as evidenced by a calibration slope >1 and a positive calibration intercept.

### Sensitivity and specificity

At both 6 months and 1 year, using a KFRE threshold of ≥40% instead of an eGFR threshold of <15 mL/min/1.73 m² resulted in a net gain in specificity with only a moderate loss in sensitivity for predicting ESKD (Table 3). The 8-variable KFRE slightly outperformed the 4-variable KFRE at both 6 months and 1 year, as indicated by superior Youden Indexes.

**Table 3:** Sensitivity, specificity and Youden Index at various times for eGFR threshold of <15 mL/min/1.73 m² and 4- and 8-variable KFRE thresholds of ≥40%.

	Sensitivity	Specificity	Youden Index
eGFR <15 mL/min/1.73 m²			
6 months	89.6	46.6	0.362
1 year	85	53.9	0.389
2 years	77.2	63	0.402
4-variable KFRE ≥40%			
6 months	81.8	67.1	0.489
1 year	75	77.4	0.524
2 years	64.5	88.7	0.532
8-variable KFRE ≥40%			
6 months	83.1	70.4	0.535
1 year	74.8	80.7	0.555
2 years	62	88.1	0.501

### Net benefit

Net benefit curves demonstrated a benefit of using KFRE over a 15%–20% threshold probability, with the 8-variable KFRE being more effective than the 4-variable KFRE at 6 months (Supplementary data, Fig. S4). The net benefit was similar between the 4- and 8-variable KFREs at 1 year (Supplementary data, Fig. S5).

## DISCUSSION

In this validation study focusing on patients with advanced CKD referred for vascular mapping, we observed that a ≥40% 2-year KFRE threshold might be more effective than a <15 mL/min/1.73 m² eGFR threshold for predicting ESKD. Therefore, it could be a useful tool for timely vascular access creation.

Our cohort predominantly comprised patients with CKD stage 5, which significantly differs from the development cohort [7] (mean eGFR 13 mL/min/1.73 m² vs 36 mL/min/1.73 m²). The median UACR was notably higher (957 mg/g vs 93 mg/g in the development cohort), attributed to a substantial prevalence of glomerular diseases (55.9% with diabetic nephropathy accounting for 39.1%).

The high incidence of ESKD (74.8%) in our study was anticipated due to its design, but the higher-than-expected death rate (9.2%) warrants discussion. Patients selected for vascular access creation were typically not in conservative care but some might have transitioned to conservative care later. Most patients started dialysis due to low eGFR as determined by their nephrologists, aligning with current best practices [20]. Our study showed a lower incidence of starting dialysis via a central venous catheter compared with the REIN Registry (29.7% vs 58%). This difference could be attributed to our study's focus on patients under nephrology care, who are presumably better prepared for dialysis.

Given the optimal window for vascular access creation is 6 to 9 months before dialysis initiation [4], we concentrated on the performance of eGFR and KFRE at 6 months and 1 year. Utilizing a ≥40% KFRE threshold was associated with a moderate decrease in sensitivity but a significant increase in specificity. This implies that a ≥40% KFRE threshold could substantially lower the risk of premature AVF creation at the cost of a slightly higher risk of starting dialysis via a central venous catheter.

The study revealed a trend toward a lower competing risk of death before ESKD in patients with a KFRE ≥40%, possibly due to the inclusion of age as a variable in the KFRE calculation. A 60-year-old man with an eGFR of 15 mL/min/1.73 m² and a UACR of 1000 mg/g would have a 2-year risk of 42.45%, compared with only 29.9% for an 80-year-old with the same eGFR and UACR. However, previous studies have highlighted the limitations of including age in KFRE, particularly for patients over 80 years, where there is a tendency to overestimate risk [21].

In our cohort, the 8-variable KFRE was the only one to demonstrate a superior predictive accuracy for 6-month ESKD compared with the 4-variable version, with better C-statistics and Net Benefit. However, this advantage disappeared when predicting 1-year ESKD. The performance of the 8-variable KFRE at 6 months might be due to its ability to identify patients with significant metabolic disorders that are challenging to correct, a key factor in deciding to initiate dialysis. Conversely, the 4-variable KFRE did not show superior discrimination to eGFR for predicting 6-month ESKD. Our study's calibration showed that the 4-variable KFRE significantly underestimated the 2-year ESKD risk in our cohort, with observed ESKD rates above 90% when the predicted probability was between 40% and 50%. This discrepancy, likely due to the high event rate, should be considered by clinicians when using KFRE to guide decisions about rapid vascular access creation.

A key strength of our study is its focus on evaluating KFRE and the ≥40% KFRE threshold in a cohort specifically undergoing vascular access creation, differing from previous studies that evaluated KFRE using registry data [8, 9]. To our knowledge, this is the first study comparing the performance of eGFR, 4-variable KFRE and 8-variable KFRE for planning vascular access, while also considering the competing risk of death. Additionally, we introduced novel markers, such as Net Benefit, to evaluate KFRE. However, our study has limitations. Due to its small sample size, the C-statistics at 6 months and 1 year between eGFR and the 4- and 8-variable KFRE are not statistically different. It is important to note that reliance on *P*-values for C-statistic analysis can be misleading, as it depends on the specific statistic used, test conservativeness, etc. [22]. Additionally, the CI for ESKD between the ≥40% KFRE threshold and the <15 mL/min/1.73 m² eGFR threshold overlap, suggesting insufficient power. We were unable to compare the net benefits of eGFR and KFRE directly, as net benefit analysis requires probabilities. Also, our study is monocentric, and the practices for creating vascular access can vary widely across centers, influenced by factors such as adherence to KDOQI guidelines [5], time to AVF creation and center-specific rates of AVF primary failure. Lastly, the retrospective nature of our study limits the strength of its conclusions. Prospective studies are essential to firmly establish the utility of KFRE and the ≥40% KFRE threshold in planning vascular access creation.

Determining the ideal timing for vascular access creation remains a complex task, with few studies exploring the application of the KFRE for this purpose. Atiquzzaman *et al*. found a benefit in adding the KFRE to the existing eGFR-based referral for vascular access creation, with 58% of patients starting on AVF within 2 years when using KFRE, compared with only 49% with an eGFR-based referral [14]. They also observed a reduction in premature AVF creation: 31% of patients with an eGFR-based referral had an AVF but did not start dialysis within 2 years, versus 18% when KFRE was added.

Their study focused on predicting effective dialysis initiation on an AVF, whereas we focused on ESKD prediction, but we found similar results. We believe that studying the benefit of KFRE on effective dialysis initiation on AVF in our retrospective study should be approached with caution due to the late referral of some patients with very low eGFR and high KFRE, for whom it would be challenging to prevent dialysis initiation on a central venous catheter, and due to the absence of information on post-AVF creation procedures.

In their study, 366 out of 1562 patients (23.4%) who started dialysis within 2 years had a KFRE ≤40%. Atiquzzaman *et al*. concluded that KFRE lacked specificity in predicting dialysis initiation. However, we believe this conclusion reflects a sensitivity issue, indicating a lower probability of having a KFRE >40% among patients who reached ESKD.

Indeed, in our study, we observed a moderate decrease in sensitivity at both 6 months and 1 year, counterbalanced by a significant increase in specificity with a ≥40% KFRE threshold.

Compared with these results, we provide additional information on the usefulness of the 8-variable KFRE and on eGFR at dialysis initiation, which is a key element for the generalizability of the results.

For predicting ESKD within a 6-month period, the 8-variable KFRE emerges as the more suitable choice. However, for forecasting 1-year ESKD, both 4-variable and 8-variable KFRE models are applicable. The decision to use one over the other may depend on factors such as operating room availability and the rate of AVF primary failure. It is crucial for clinicians to recognize that the 2-year KFRE is markedly undercalibrated in this specific patient population. Consequently, while it provides valuable insights, the KFRE should be interpreted with caution and in the context of other clinical considerations.

## CONCLUSION

This study reinforces the upcoming KDIGO guidelines for CKD evaluation and management, which advocate for a ≥40% 2-year threshold in planning vascular access creation. Implementing this threshold could potentially mitigate the risk of premature AVF creation, though it does slightly elevate the risk of initiating dialysis via a central venous catheter.

This balanced approach can assist in optimizing patient outcomes in the context of complex clinical decision-making.

## Supplementary Material

sfae220_Supplemental_File

## Data Availability

The data underlying this article will be shared on reasonable request to the corresponding author.
