# Supplementary material for: Enhancing vascular access planning in CKD: validating the 40% KFRE threshold for predicting ESKD in a French retrospective cohort study
Source: Clin Kidney J. 2024 Jul 13;17(8):sfae220. doi: 10.1093/ckj/sfae220 (PMC11483627; doi:10.1093/ckj/sfae220)

**Supplemental Figures and Tables**

**Supplemental Figure 1: Cumulative Incidence Functions (CIF) for Death and ESKD: Comparison Based on 8-Variable KFRE Thresholds (< vs ≥40%)**

**Supplemental Figure 2: ROC Curve Analysis and C-Statistics for 2-Year ESKD Prediction: Comparing eGFR, 4-Variable KFRE, and 8-Variable KFRE**

**Supplemental Figure 3: Calibration Curve for 4-Variable KFRE in Predicting 2-Year Risk of ESKD**

**Supplemental Figure 4: Decision Analysis of Net Benefit at 6 Months: Evaluating Threshold Probabilities for 4-Variable and 8-Variable KFRE**

**Supplemental Figure 5: Decision Analysis of Net Benefit at 1 Year: Comparative Threshold Probability Assessment for 4-Variable and 8-Variable KFRE**

**Supplemental Figure 1: Cumulative Incidence Functions (CIF) for Death and ESKD: Comparison Based on 8-Variable KFRE Thresholds (< vs ≥ 40%).** If 8 variables KFRE was ≥40%, probability of ESKD at 6 months was 57.7% (48.4-66.9%), 80.2% (72.7-87.7%) at 12 months and 93% (87.6-98.4 %) at 24 months, probability of competing risk of death was 1.2% (0-3.6%) at 24 months. If 8 Variables KFRE was < 40%, probability of competing risk of death was 8.5% at 24 months (4.8-16.2%)

**
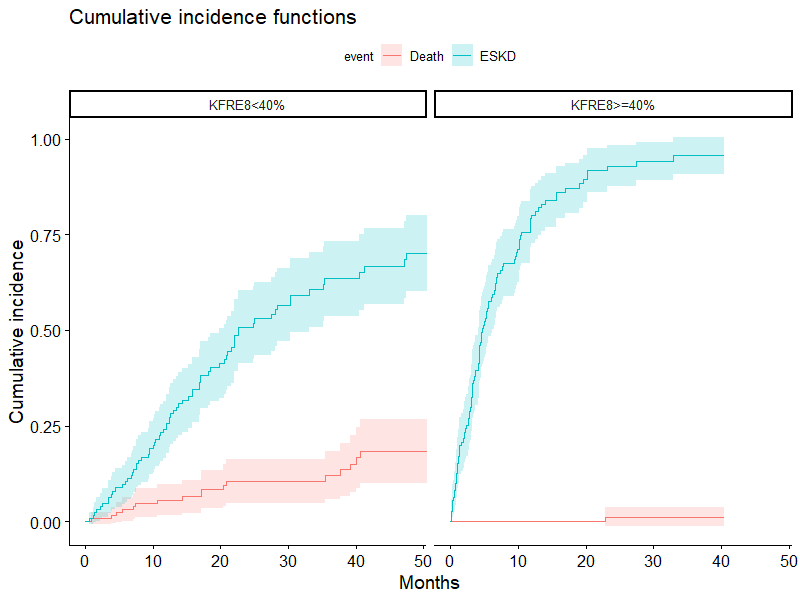
**

**Supplemental Figure 2 : ROC Curve Analysis and C-Statistics for 2-Year ESKD Prediction: Comparing eGFR, 4-Variable KFRE, and 8-Variable KFRE**

**
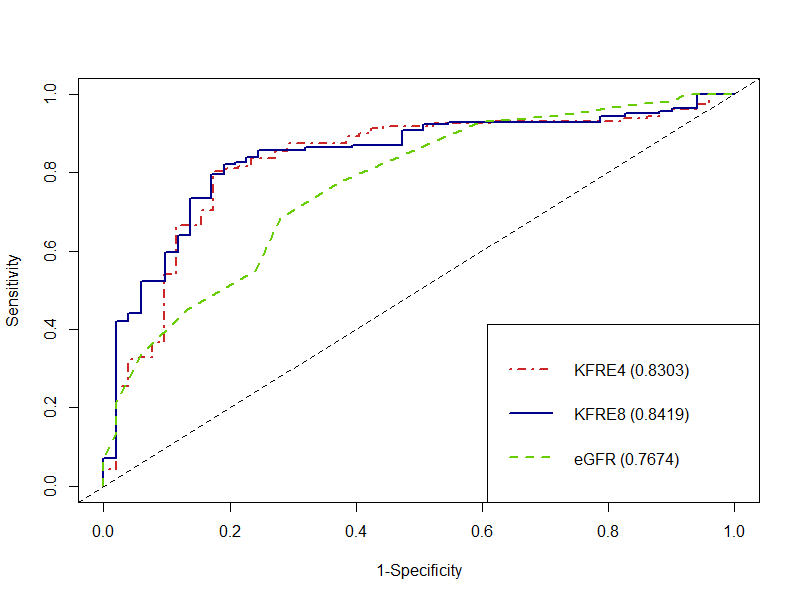
**

**Supplemental Figure 3 : Calibration Curve for 4-Variable KFRE in Predicting 2-Year Risk of ESKD**


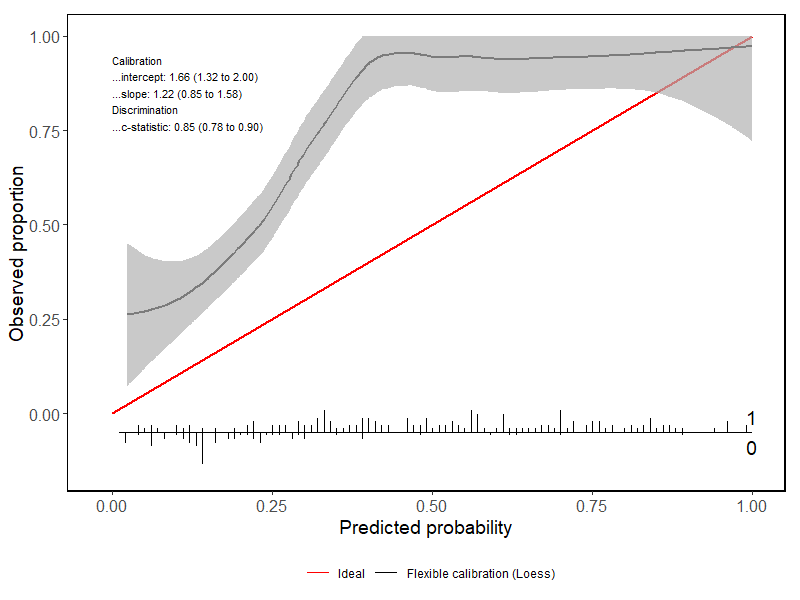


In this calibration curve, the relationship between observed and predicted probabilities of the outcome is described graphically. Plotting the smoothed regression line allows one to examine calibration across the range of predicted values and to determine if there are segments of the range in which the model is poorly calibrated.

In this cohort, 4 variables KFRE was clearly undercalibrated for predicting 2-year risk of ESKD with an observed proportion of ESKD above 90% when the predicted probability is above 40-50%.

**Supplemental Figure 4 : Decision Analysis of Net Benefit at 6 Months: Evaluating Threshold Probabilities for 4-Variable and 8-Variable KFRE.** In this decision analysis, the concept of net benefit is analogous to an economic balance, where the gain from true positives (akin to income) is weighed against the cost of false positives (akin to expenditure), and the "exchange rate" is defined by the acceptable number of false positives per true positive. The "treat all" baseline represents the net benefit of universally applying the intervention (in this case, the creation of an Arteriovenous Fistula, or AVF), while the "treat none" baseline represents the net benefit of withholding the intervention across the board. The analysis reveals that when the threshold probability exceeds 15%, both the 4-variable and 8-variable KFRE outperform the strategy of treating all patients. Notably, the 8-variable KFRE demonstrates a greater net benefit than the 4-variable KFRE, indicating a more advantageous trade-off between true and false positives at this threshold**.**


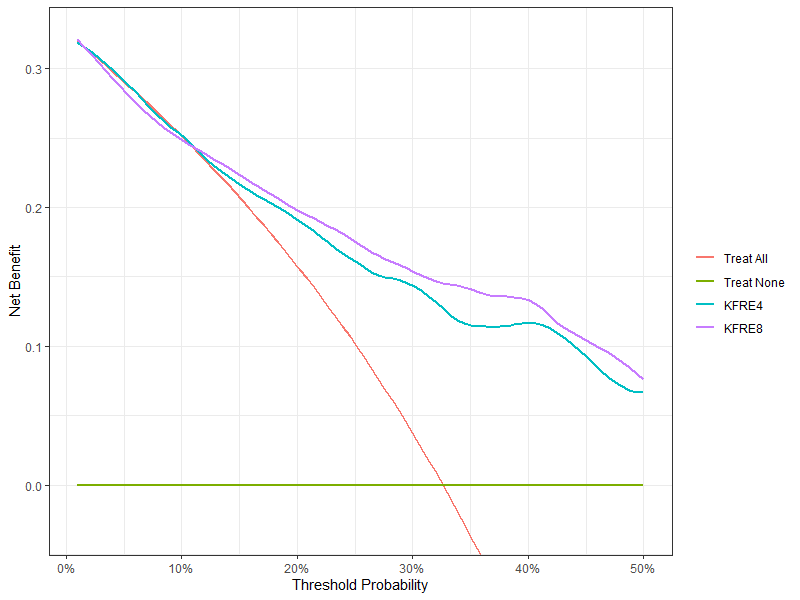


**Supplemental Figure 5 : Decision Analysis of Net Benefit at 1 Year: Comparative Threshold Probability Assessment for 4-Variable and 8-Variable KFRE.** In this decision analysis, the concept of net benefit is analogous to an economic balance, where the gain from true positives (akin to income) is weighed against the cost of false positives (akin to expenditure), and the "exchange rate" is defined by the acceptable number of false positives per true positive. The "treat all" baseline represents the net benefit of universally applying the intervention (in this case, the creation of an Arteriovenous Fistula, or AVF), while the "treat none" baseline represents the net benefit of withholding the intervention across the board.


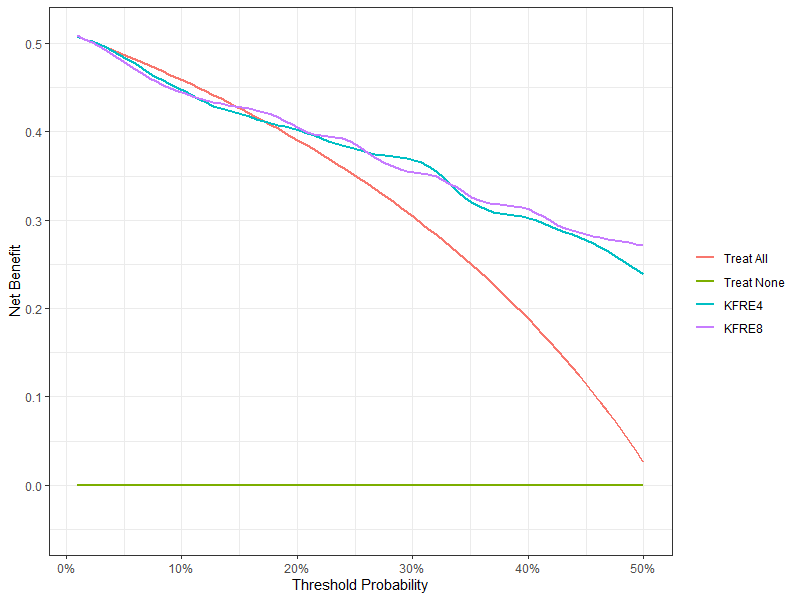

Supplement: sfae220_Supplemental_File [file sfae220_Supplemental_File.docx]
